# Episodic memory training in elderly: A systematic review

**DOI:** 10.3389/fpsyg.2022.947519

**Published:** 2022-07-28

**Authors:** André Rocha Mendonça, Lucas Machado Loureiro, Carlos Eduardo Nórte, Jesus Landeira-Fernandez

**Affiliations:** ^1^Department of Psychology, Pontifical Catholic University of Rio de Janeiro, Rio de Janeiro, Brazil; ^2^Institute of Psychology, State University of Rio de Janeiro, Rio de Janeiro, Brazil

**Keywords:** episodic memory, elderly, cognitive training, neuropsychology, systematic review

## Abstract

**Introduction:**

Episodic memory is a cognitive process that allows the recall of experiences, learning, and the pursuit of future goals. During the aging process, episodic memory declines negatively, impacting social and psychological aspects in the elderly. Such intervention strategies as cognitive training are non-pharmacological ways of reducing these losses.

**Objective:**

We systematically reviewed studies of the cognitive training of episodic memory in healthy elderly individuals and elderly individuals with clinical conditions.

**Method:**

We systematically searched the PubMed, PsycNET, Web of Science, and SciELO databases using the descriptors “Episodic Memory” AND “Training” AND “Elderly” OR “Aging” OR “Dementia” in English and translated into Portuguese.

**Results:**

Of the 572 articles that were identified by the search, 23 were included in the final analysis. The main variables that comprised the intervention protocols (i.e., characteristics of the sample, type of control group, mode of episodic memory training, and duration of training) were investigated, as well as the outcome variables, efficacy, and risk of bias. The main results indicated effectiveness with regard to different forms of the cognitive training of episodic memory.

**Conclusion:**

Episodic memory training among the elderly can mitigate the negative effects of cognitive decline in dementia and healthy subjects promoting impacts at social, psychological, and economic levels. Although it is a widely studied topic, further empirical studies on the utility of episodic memory training in healthy elderly individuals and elderly individuals with clinical conditions are still needed.

## Introduction

Episodic memory is a form of memory that allows the recall of events to enable learning and the pursuit of future goals based on an individual's experiences. According to Tulving (2002), episodic memory permits an individual to subjectively move through time (i.e., travel through his own self toward the past) while knowing that he is accessing remote experiences that were previously recorded. Thus, episodic memory has an explicit nature, and its content is consciously accessed. Additionally, the storage of information is long term and remains for an indefinite period of time, from days to decades. However, the total capacity of episodic memory to store information is still unknown.

Many studies of episodic memory have investigated interactions with cognitive resources, emotional regulation, environmental adaptation, attention, language, and executive function (Bahar-Fuchs et al., 2019). Episodic memory is related to autobiographical memory; both share a record of events based on place or time, thereby influencing the notion of self (Tulving, 2002). Affect also participates in the modulation of an event that is to be consolidated in episodic memory. Emotionally charged events are easier to record than others. The emotional state also influences the evocation of memories. The current affective state provides contextual mnemonic cues to evoke recorded information that is compatible with the experienced moment (da Costa Pinto, 2003; Pergher et al., 2006).

Higher life expectancies produce demands with regard to care that a given society provides for its elderly population (Assed et al., 2016). Studies of impacts on the physical and mental health of this population are increasing, especially with regard to deficits in episodic memory and their consequences on the lives of the elderly. These deficits result from impairments in the evocation of and access to mnemonic records, in addition to the lesser use of coding and storage strategies for new information, which may reflect natural senescence or cognitive decline (Aramaki and Yassuda, 2011). These mnemonic difficulties impact the lives of the elderly and can compromise daily function, such as remembering or associating information, managing finances, handling and controlling medications, orientating in time and space, and having autonomy in traveling outside the home to go shopping or enjoying leisure activities (Fandakova et al., 2012; Giovagnoli et al., 2017).

Among intervention strategies that have been developed to address episodic memory deficits, whether caused by senescence or psychopathological conditions, cognitive training has been the most used for the elderly (Bahar-Fuchs et al., 2013). Episodic memory training consists of exercises to reduce cognitive deficits and improve certain skills, thus providing healthier aging and maintaining an individual's daily function (Banducci et al., 2017). This type of non-pharmacological intervention employs systematic activities that focus on specific mental functions that seek to strengthen certain areas of cognition and meet specific objectives (Aramaki and Yassuda, 2011). Cognitive training can be structured individually or in groups. It usually occurs over a period of time. Its effects reflect behavioral and cognitive changes, measured by neuropsychological tests, the neurobiological base of which is anchored in cortical plasticity (Apóstolo et al., 2011; Nousia et al., 2018).

Controlled studies have reported favorable effects of cognitive training on a wide range of cognitive functions, such as attention, memory, processing speed, language, planning skills, and different problem-solving strategies, with positive effects in healthy elderly individuals (Chambon et al., 2014). Improvements were also found in such aspects as mood and well-being in patients with mild cognitive impairment (MCI) and Alzheimer's disease (AD; Kurz et al., 2008). However, Chambon et al. (2014) reported that memory performance can be stimulated not only by establishing mnemonic strategies to sustain the processes of learning, autonomy, and well-being but also by reducing negative beliefs about memory in aging.

The mapping of non-pharmacological methods of intervention is necessary to reduce the impact of dementia, ameliorate the negative impacts of senescence on memory, and manage practical issues about daily function and daily demands that burden caregivers. A meta-analysis by Floyd and Scogin (1997) was one of the first studies in this domain that sought to map the effectiveness of memory training in subjective memory function and mental health in the elderly. The authors stated that the uplift of subjective memory function was made even better by including pretraining that involved skills like the use of images and interventions to improve the attitudes of the participants. In the Brazilian context, a systematic review by Santos and Flores-Mendoza (2017) provided an overview of the Brazilian literature on cognitive training for the elderly, highlighting the trend in group interventions, instead of individual ones, that seek to stimulate episodic memory.

Another systematic review by Mendes et al. (2019) considered studies of cognitive stimulation in groups of elderly individuals. The studies they found tended to corroborate the idea that cognitive stimulation among groups is a good strategy for health professionals who attempt to preserve cognitive skills and prevent their decline in the elderly. Their work not only reported an efficacy of this type of intervention in cognitive training, but also extended its benefits to the mood and socialization of individuals, contributing to a greater quality of life in general.

A further recent systematic review on the topic by Bahar-Fuchs et al. (2019) expanded the mapping of the effects of cognitive training by investigating various cognitive domains in elderly individuals with mild to moderate dementia. Their main results indicated that cognitive training has small to moderate positive effects on global cognition, and these gains can be maintained for 3–12 months after intervention when compared with a control group. Despite existing reviews, several empirical questions about training episodic memory in the elderly remain unanswered.

## Objective

The present study systematically reviewed studies on the application of cognitive training for episodic memory in healthy elderly individuals and elderly individuals with clinical conditions, including MCI and AD. We investigated (1) sample characteristics (i.e., sex and age), (2) the existence of a control group and its profile (i.e., active or passive), (3) types of episodic memory training that were used (i.e., duration and stimuli), (4) the protocols that were used and their outcome variables, (5) methodological quality and risk of bias in the studies, (6) effectiveness of the intervention, and (7) effects of the intervention at follow-up.

## Method

We used the Preferred Reporting Items for Systematic Reviews and Meta-Analyzes (PRISMA) guidelines (Moher et al., 2009). The PRISMA method is based on a protocol that was developed in a meeting with 29 participants, including clinicians, researchers, and editors, that established criteria for conducting systematic reviews and meta-analyses.

According to Moher et al. (2009), the construction of the review should consist of the following steps: (1) group articles that are found in the databases, removing possible duplicates, (2) exclude articles after an initial evaluation of the titles, (3) review the abstracts of the remaining studies according to previously established criteria, (4) analyze the remaining studies in full to verify that they meet the eligibility criteria, and (5) extract and systematize the variables of interest in the included studies.

### Study selection

The studies that were identified in the literature search were chosen according to the following inclusion criteria: (1) articles in English or Portuguese, (2) intervention based on episodic memory training, (3) subjects >60 years of age, (4) healthy elderly individuals or elderly individuals with some clinical condition, (5) cohort studies, and (6) outcome variables that were measured using instruments that objectively assess episodic memory. The exclusion criteria were the following: (1) case studies, (2) animal studies, (3) theoretical works, and (4) studies that did not objectively measure episodic memory.

### Search strategy

Four databases were searched: PubMed; PsycNET, Web of Science, and SciELO. The searches were performed on December 11, 2019, using the following keywords: “Episodic Memory” AND “Training” AND “Elderly” OR “Aging” OR “Dementia.” We also searched the Portuguese words for these search terms, which were exclusively used in the SciELO database. The screening steps for resulting articles were performed and discussed by two authors of this study.

## Results

**Figure 1:**
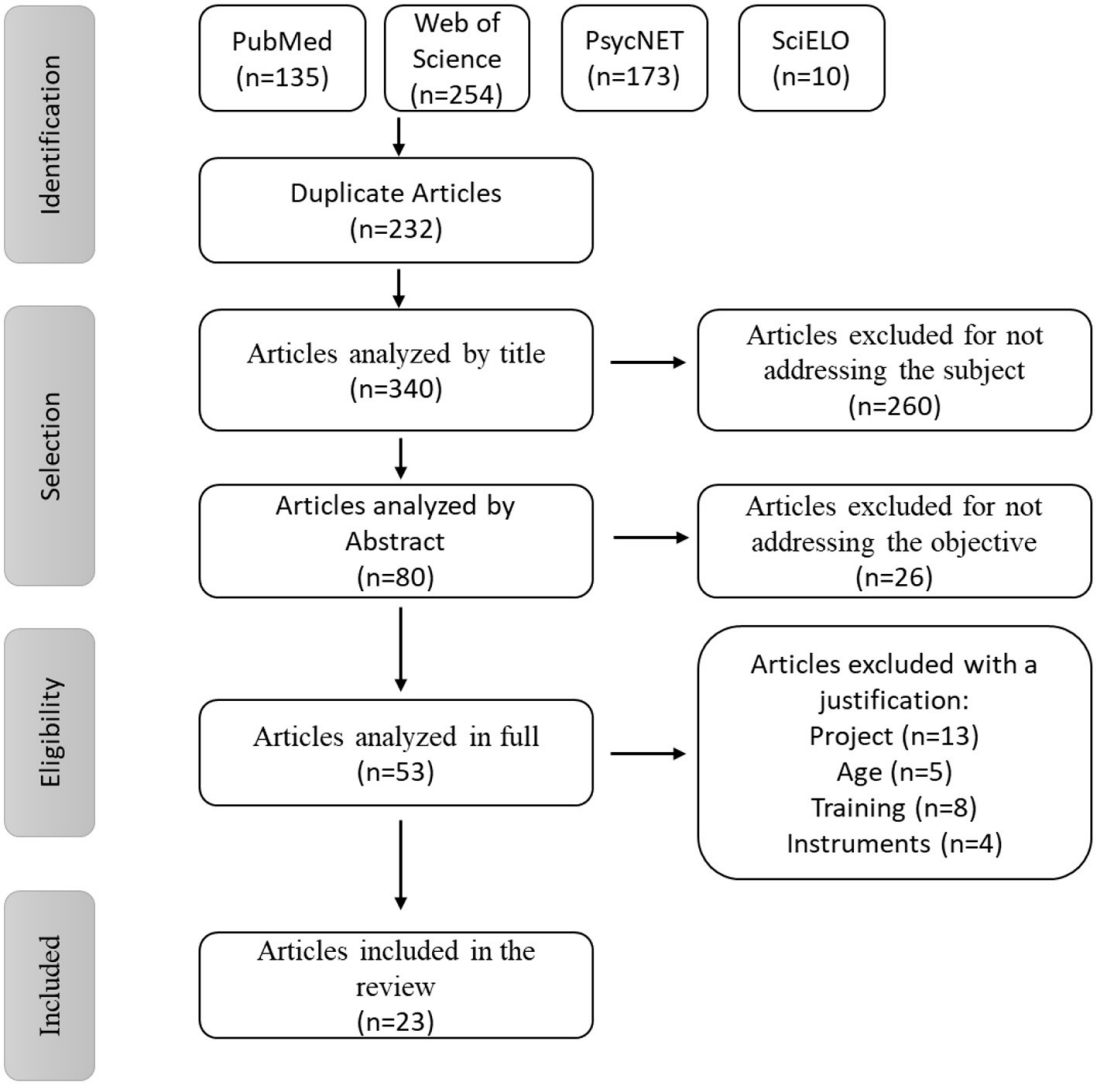
PRISMA flowchart adapted from Moher et al. (2009).

Using the PRISMA method (Moher et al., 2009), the search yielded 572 articles in PubMed (135), Web of Science (254), PsycNET (173), and SciELO (10). Of these 572 articles, 340 were eligible after eliminating duplicate articles and performing the screening steps based on the title, abstract, and full text of the articles. According to the inclusion and exclusion criteria, 23 articles were subjected to data collection and analysis. Figure 1 summarizes the study selection process.

## Characteristics of the studies

### Subjects and clinical conditions

Table 1 depicts the characteristics of the studies that were included in the present review. The number of participants who were recruited varied in the 23 studies that employed various models of cognitive intervention. The smallest group consisted of 16 participants (Aramaki and Yassuda, 2011), and the largest consisted of 2,802 participants (Willis et al., 2006), with an average of 392.57 participants per study (standard deviation = 787.628, standard error = 164.238, median = 57). The subjects were 60–89 years of age and their education ranged from 0–2 years (da Silva and Yassuda, 2009) to 16 years (Banducci et al., 2017; Savulich et al., 2017). In a large proportion of the studies, women represented the majority of the participants. With regard to the cognitive conditions of the different groups, 13 studies (56.52%) evaluated healthy elderly individuals (Ball et al., 2002; Willis et al., 2006; Carvalho et al., 2009; da Silva and Yassuda, 2009; Langbaum et al., 2009; Lima-Silva et al., 2010; Aramaki and Yassuda, 2011; Gross and Rebok, 2011; Legault et al., 2011; Fandakova et al., 2012; Chambon et al., 2014; Zimmermann et al., 2016; Banducci et al., 2017), six studies (26.09%) investigated elderly individuals with clinical conditions (either MCI or AD; Kurz et al., 2008; Jean et al., 2014; Neely et al., 2014; Giovagnoli et al., 2017; Savulich et al., 2017; Nousia et al., 2018), and four studies (17.39%) analyzed groups with both conditions (Belleville et al., 2006; González-Palau et al., 2014; Kinsella et al., 2016; McDougall et al., 2018).

**Table 1:** Characteristics of the studies that were included in the systematic review.

**Reference**	**Participants and conditions**	**Comparison groups**	**Intervention and duration**	**Measures of episodic memory**	**Efficacy**
Banducci et al. (2017)	*n* = 179	Active control	Active Control. This type of training seeks to stimulate through theater using scripts and promote social interaction between participants	Logical memory (Virginia Cognitive Aging Project)	Significant
	Healthy	*n* = 86	75-min group training twice per week for 4 weeks		
Nousia et al. (2018)	*n* = 50	Passive control	Training was divided into two parts. The first part used a computer in 30-min sessions to stimulate mainly such activities as memory, attention and processing speed. The second part stimulated mostly language using a pen and paper	Task with repetition and word recognition delayed memory	Significant
	Alzheimer's disease early stage	*n* = 25	Individual training with 60-min sessions over 15 weeks		
Chambon et al. (2014)	*n* = 45	Active and passive control	Training using a computer with tasks that stimulate attention, memory (short-term, working, visuospatial, and narrative)	Doors and People visual recognition test; 16-Item Free Reminding Test (RL/RI-16 Test); 12-word recall test from the BEM-144 memory battery	Significant
	Healthy	*n* = 30	One-hour individual session per day, twice per week, for a total of 24 sessions		
Kurz et al. (2008)	*n* = 40	Active and passive control	Similar to a workshop with activities that seek to stimulate skills in distinguishing relevant and irrelevant information using mnemonic strategies. Training included motor skills, communication skills, and social interaction, in addition to practicing manual work, leisure activities, and relaxation	California Verbal Learning Test; Rey Complex Figure	Significant
	MCI and dementia early stage	*n* = 12	Group activities (10 members/group), 6 h per day on weekdays over 4 weeks		
Aramaki and Yassuda (2011)	*n* = 16	No control group	Training with mental images as resources for memorizing words, phrases, and tales, in addition to psychoeducational sessions to change negative beliefs regarding memory	Brief Cognitive Screening Battery	Significant
	Healthy		Activities performed with the same group over five sessions, 45 min each. The first part occurred in the second half of 2008, and the second part occurred 18 months later	Mini Mental State Examination; Figure Memory	
Giovagnoli et al. (2017)	*n* = 50	Active control	Cognitive training with exercises that involve attention, information processing, executive function, and memory	Short Story Test; Rey Auditory-Verbal Learning; Rey Complex Figure	Significant
	Alzheimer's disease early stage	*n* = 33	45-min group activity for 12 weeks over 3 months		
Savulich et al. (2017)	*n* = 42	Passive control	Games with attractive screens and stimulating music. According to the studies, the aim was to improve episodic memory capacity	Paired Associates Learning CANTAB PAL; Mini Mental State Examination	Significant
	MCI amnestic	*n* = 21	Group activities of 1 h each for a total of eight sessions		
Neely et al. (2014)	*n* = 60	Active and passive control	Collaborative program in which the spouse with dementia and the caregiver received assistance to support memory performance and everyday occupational tasks	Use of ecological tasks that measure episodic recall capabilities and the capacity of categorizing previously memorized objects	Significant
	Mild to moderate vascular dementia or Alzheimer's disease	*n* = 40	Individual activities that lasted 1 h, once per week, for a total of eight sessions		
Legault et al. (2011)	*n* = 73	Control group	Cognitive training using computers with four consecutive exercises. In each session, the participants studied a list of 30 words and had to subsequently recognize the 30 memorized words among 30 new words. The training group was based on physical activities, such as aerobics and flexibility. The control group was part of the Aging Education Program from Lifestyle Interventions. The study included groups of up to seven people and lasted according to the training: cognitive training (eight sessions over 4 months), physical training (32 sessions over 4 months), combined training (56 sessions, with 24 sessions for cognitive training and 32 sessions for physical training)	Hopkins Verbal Learning Test; Logical Memory I and II (Wechsler Memory Scale-III memory task)	Significant
	Healthy	*n* = 19			
Lima-Silva et al. (2010)	*n* = 69	Passive control	Training began with an educational intervention to eliminate negative beliefs about aging-related memory decline. Afterward, tasks were applied to memorize words, phrases, and stories	Brief Cognitive Screening Battery; Figure memory	Significant
	Healthy	*n* = 32	Five group sessions, 45 min each		
Ball et al. (2002)	*n* = 2082	Active and passive control *n* = 698	Training with initial sessions (1–5) focused on practicing strategies. In the remaining sessions (6–10), additional exercises were practiced but without new strategies. The strategies sought to improve the ability to memorize lists, sequences of words and items, texts, and details of stories	Learning Hopkins Verbal Learning Test; Rey Auditory-Verbal	Significant
	Healthy		Group training with 10 sessions, 6–75 min each, over 5–6 weeks		
Jean et al. (2014)	*n* = 22	Active control	Training sought to encourage participating individuals to relearn through the association of names (first and last names) among five unknown and famous individuals within the artistic, political, scientific, and sports fields. In addition to mnemonic training, pedagogical content was given to provide more information regarding memory	California Verbal Learning Test, 2nd edition	Significant
	MCI with single or multiple domain	*n* = 11	Individual 45-min sessions, twice per week, for 3 weeks		
Carvalho et al. (2009)	*n* = 57	Passive control	Training sought to stimulate categorization strategies using techniques that utilized visual and/or verbal material. There were stages in which the participants performed the categorization at home and then in the classroom. In other sessions, they performed the categorization on the day of the session without having had contact with the material to be categorized	Episodic Memory Task, in which the individual being evaluated memorized two boards with figures. The individual was then subjected to a distracting activity; afterward, the individual had to evoke the figures that were previously seen	Significant
	Healthy	*n* = 26	Five group sessions (13–23 subjects/group) that lasted 1 h twice per week		
Fandakova et al. (2012)	*n* = 42	Active control	Use of associative recognition	Tasks of name-face associations and recall memory	Significant
	Healthy	*n* = 42	Group sessions with no specified duration		
Belleville et al. (2006)	*n* = 47	Active and passive control *n* = 16	Attention training using a computer, with split-attention training, visual detection, arithmetic tasks, and the memorization of specific places at home and later their association with words to form mental maps. Text hierarchization, learning, and verbal organization were trained according to semantic proximity and categorization	Name-face recall tasks and learning a list of unrelated words	Significant
	Healthy and MCI		Eight weekly sessions that lasted 2 h each		
Willis et al. (2006)	*n* = 2802 Healthy	Active and passive control *n* = 698	Cognitive training using mnemonic strategies (organization, visualization, and association) to recall verbal material, such as lists of words and texts, in addition to training reasoning, processing speed, and split-attention strategies	Hopkins Verbal Learning Test; Rey Auditory-Verbal Learning; Rivermead Behavioral Test–Paragraph Recall	Significant
			Ten 1-h group sessions with initial training plus four 1-h sessions after 11 and 35 months		
Gross and Rebok (2011)	*n* = 210	Active and passive control *n* = 698	Intervention with cognitive training, reasoning, information processing speed, and strategies for better use of memory	Hopkins Verbal Learning Test; Rey Auditory-Verbal Learning; Rivermead Behavioral Test–Paragraph Recall	Significant
	Healthy		Ten 1-h sessions over 6 weeks		
McDougall et al. (2018)	*n* = 263	Active control	Training sessions with relaxation practice and activities to be performed at home and discussed in the next session. Throughout the sessions, memory was trained with 30 min of practice and strategies to strengthen memory skills. After each session, the participants wrote aspects of their learning or essential points, such as a recall strategy	Hopkins Verbal Learning Test; Rivermead Behavioral Test–Paragraph Recall; questionnaire on subjective memory complaints	Significant
	Healthy and with MCI	*n* = 20	Eight group sessions twice per week and four additional sessions once per week over 3 months in the post-test		
da Silva and Yassuda (2009)	*n* = 29	Active control	Training was based on a Category group (CATG) and Mental Image group (IMG):	Rivermead Behavioral Test–Paragraph Recall; visual episodic memory test (18 figures); auditory memory test with recall of a story; assessment of memorizing strategies based on self-report as a measure of Bousfield categorization	Significant
	Healthy	*n* = 13	CATG group: Exercises with visual and auditory attention, verbal fluency, and episodic memory training; episodic memory tasks involved memorizing items found in a grocery store, photos, and figures. Participants were suggested to categorize objects and figures according to function, color, or shape		
			IMG Group: Creation of mental images of individual items of various objects found in a supermarket. They were also encouraged to imagine such items in different conditions that involved movement, color, smell, and spatial dispositions		
			Eight group sessions that lasted 1 h 30 min each, twice per week		
Langbaum et al. (2009)	*n* = 619	Passive control	The experimental group participated in training memory, inductive reasoning, and processing speed. The control group participated in activities that sought to improve mental skills and activities of daily life	Hopkins Verbal Learning Test;	Significant
	Healthy	*n* = 64	Group sessions that lasted 10 weeks	Rey Auditory-Verbal Learning; Rivermead Behavioral Test–Paragraph Recall	
Kinsella et al. (2016)	*n* = 219	Passive control	Interactive sessions to convey information about memory, its changes (disorders), and repercussions related to quality of life. Use of cognitive strategies, such as organization, semantic association, images, and retrieval strategies	California Verbal Learning Test, 2nd edition	Significant
	Healthy and MCI	*n* = 106	Six 2-h group sessions per week	Prolonged Delayed Evocation Task;	
				Logical memory (Delayed recall);	
				Wechsler Memory scale subtest;	
				Paired verbal association;	
				Rey Complex Figure	
González-Palau et al. (2014)	*n* = 50	Active control	Computer with Gradiator software. This software was designed as a training system that included activities of attention, perception, episodic memory, and working memory	Hopkins Verbal Learning Test, Revised	Significant
	Healthy and with MCI	*n* = 11	Individual 40-min sessions, three times per week, for 12 weeks		
Zimmermann et al. (2016)	*n* = 67	Active control	Tasks aimed at locating objects and landmarks	Use of prospective subtasks	Significant
	Healthy	*n* = 36	Fifteen sessions that were held in two phases, separated by 1 week. Use of groups of up to four participants	Berlin 4 intelligence test, paper and pencil form; Computerized tasks	

The participants were recruited from geriatric outpatient clinics or psychiatric institutions (Belleville et al., 2006; Kurz et al., 2008; Jean et al., 2014; Neely et al., 2014; Savulich et al., 2017; Nousia et al., 2018), community centers (Ball et al., 2002; Willis et al., 2006; Carvalho et al., 2009; Legault et al., 2011; Fandakova et al., 2012; González-Palau et al., 2014; Kinsella et al., 2016; Banducci et al., 2017; Giovagnoli et al., 2017; McDougall et al., 2018), schools and colleges for the elderly (da Silva and Yassuda, 2009; Lima-Silva et al., 2010; Aramaki and Yassuda, 2011; González-Palau et al., 2014), or home visits (Langbaum et al., 2009; Chambon et al., 2014). The elderly participants volunteered to undergo different forms of interventions. The majority of them underwent a screening stage that used brief screening batteries to assess the inclusion and exclusion criteria and control for confounding variables.

### Comparison groups

Most of the studies (95.65%) included a control group. However, in a comparative study of two interventions, with an interval of 18 months between interventions (Aramaki and Yassuda, 2011), this was not possible because the control group that participated in the first intervention was stimulated during the interval between interventions, which prevented the reuse of the control group in the second intervention.

In 16 studies (69.57%), half (34.78%) used only an active control group, and the other half used only a passive control group. The remaining six studies (26.09%) included a control group with both conditions (active and passive).

### Type of cognitive training

The structure of the stimulation programs and cognitive processes that were involved were similar, which focused mainly on attentional capacities, processing speed, and memory. The strategies for the stimulation of these skills, mostly episodic memory, involved physical exercises, the use of computers, and psychopedagogical sessions that sought to clarify doubts or negative beliefs about memory decline that is common in old age (Aramaki and Yassuda, 2011; Jean et al., 2014). The articles that were reviewed herein were categorized according to the type of training.

Neely et al. (2014) developed a collaborative stimulation program, in which the spouse or caregiver assisted the partner with dementia in tasks that require better episodic memory performance and daily occupational tasks to support activities of categorization and memorization. Legault et al. (2011) used a method that involved the integration of cognitive training with physical exercise. González-Palau et al. (2014) implemented the same method but included computers as a tool for cognitive training.

In addition to these three studies (13.04%), another four studies (17.39%) used computers as a tool for cognitive training, with applications that stimulated attentional processes, mainly episodic memory (Chambon et al., 2014; González-Palau et al., 2014; Savulich et al., 2017; Nousia et al., 2018). Three studies (13.04%) used cognitive training based on the Advanced Cognitive Training for Independent and Vital Elderly (ACTIVE) program. In this model, the elderly participants underwent training that focused on strategies to achieve better memory, reasoning, and processing speed (Ball et al., 2002; Langbaum et al., 2009; Gross and Rebok, 2011).

The remaining 13 studies (56.52%) used conventional models to stimulate various cognitive processes, such as memory stimulation and name-face association (Jean et al., 2014) and associative recognition and the memorization and location of objects at specific points (Fandakova et al., 2012; Zimmermann et al., 2016). In addition to memory strategies, some studies focused on motor skills and relaxation exercises (Kurz et al., 2008; McDougall et al., 2018) and included psychoeducational sessions to reduce negative beliefs about memory in old age (Lima-Silva et al., 2010; Aramaki and Yassuda, 2011). Memory training also included other stimuli, such as theater scripts, and focused on attention, information processing, the memorization of words, phrases, and texts, and the categorization of previously learned information (Willis et al., 2006; Carvalho et al., 2009; da Silva and Yassuda, 2009; Kinsella et al., 2016; Banducci et al., 2017; Giovagnoli et al., 2017).

### Intervention length

In addition to different training protocols, the durations of the interventions were also heterogeneous. The interventions included a maximum of 36 sessions and minimum of five sessions (mean = 11.05 sessions, standard deviation = 7.28 sessions, mode = 10 sessions.

Fandakova et al. (2012) was the only study that did not stipulate a specific number or duration of the sessions. The study by Legault et al. (2011) was not included in the overall session count because it involved three different groups, and each group underwent a different number of sessions.

In 18 studies (78.26%), the intervention was applied in a group setting. In five studies (21.74%), the intervention was applied individually. Among the studies that reported the intervention duration, the minimum was 1 month (Kurz et al., 2008; Gross and Rebok, 2011). The training period in Giovagnoli et al. (2017) and Nousia et al. (2018) was 3 months, whereas Willis et al. (2006) and Jean et al. (2014) had the longest durations, ranging from 12 to 35 months. The length of each session also varied. Kurz et al. (2008) reported 6 h (360 min) per week. The minimum session length was 40 min (González-Palau et al., 2014). Seven studies (31.82%) had a session length of 60 min. Two studies (9.09%) had a session length of 120 min. Four studies (18.18%) had a session length of 40 min. Two studies (9.09%) had session lengths of 90 and 75 min, respectively, and four studies (18.18%) had a session length of 45 min. The study by Ball et al. (2002) was not included in this analysis because its session durations ranged from 60 to 75 mi.

Of the 23 studies, nine (39.13%) included a follow up after the post-evaluation stage. Jean et al. (2014) had a follow-up period of 1–4 weeks. Zimmermann et al. (2016) had a follow-up period of 4 months. Chambon et al. (2014) and Kinsella et al. (2016) had a follow-up period of 6 months. Willis et al. (2006) had a follow-up period of 1 year. Ball et al. (2002) and McDougall et al. (2018) had a follow-up period of 2 years. Langbaum et al. (2009) and Gross and Rebok (2011) had follow-up periods of 1, 2, 3, and 5 years after the intervervention.

### Instruments to measure episodic memory

A total of 33 different instruments were used, including various tests, tasks, and questionnaires, to measure episodic memory and the participants' performance in pre- and post-intervention stages. Three instruments (9.09%; Rey Auditory-Verbal Learning Test [RAVLT], Rivermead Behavioral Memory Test–Paragraph Recall, and Hopkins Verbal Learning Test [HVLT], which is analogous to the RAVLT) were used in five studies (21.74%). Rey's Complex Figure, representing (3.03%) of the instruments that were used, was applied in three studies (13.04%). The Brief Cognitive Screening Battery (BCSB), Picture Memory, Mini Mental State Examination (MMSE), Verbal Hopkins Learning Task, name-face association task, recall memory, word list, and California Verbal Learning Test, 2nd edition (CVLT-II), represented 18.18% of the instruments that were used and were further described by two studies (8.70%). The remaining 23 instruments (69.70%) were applied at least once in the remaining 13 studies (56.52%). Notably, this analysis did not consider the use of a given instrument more than once in the same study but rather its frequency of use relative to the total number of studies.

### Effectiveness

The analysis of effectiveness of the interventions was based on comparative performance in the experimental and control groups, which was grounded in training episodic memory and evaluations in the post-training phase. All 23 studies reported efficacy according to their own cognitive training program, but the studies compared the experimental and control groups in different ways. Thirteen studies (56.52%) performed a standard comparison [i.e., they compared post-training results between an experimental group and a control group; Belleville et al. (2006); Carvalho et al. (2009); da Silva and Yassuda (2009); Langbaum et al. (2009); Lima-Silva et al. (2010); González-Palau et al. (2014); Jean et al. (2014); Kinsella et al. (2016); Zimmermann et al. (2016); Banducci et al. (2017); Savulich et al. (2017); McDougall et al. (2018); Nousia et al. (2018)]. Five studies (21.74%) performed comparisons between experimental and control groups and further divided the control groups into passive and active conditions. The active subgroup was further divided into two additional groups according to the cognitive function that was trained (Ball et al., 2002; Gross and Rebok, 2011; Legault et al., 2011; Fandakova et al., 2012; Giovagnoli et al., 2017). Four studies (17.39%) compared the results using a control group, subdividing in active and passive control conditions (Willis et al., 2006; Kurz et al., 2008; Chambon et al., 2014; Neely et al., 2014). Only one study (4.35%) evaluated the experimental group without any control group (Aramaki and Yassuda, 2011).

### Quality of the studies

The quality of the studies was analyzed using the Newcastle-Ottawa Scale (Wells et al., 2013). This scale uses three parameters (selection, comparability, and outcome), which are subdivided into specific questions that assign points to the quality of the study. The score ranges from zero (lowest quality) to 9 (highest quality). The analysis was performed independently by two researchers, and the final scores were established by consensus. Studies with a final score ≤ 5 were considered low quality. Studies with a final score >5 were considered high quality.

Only three papers (13.04%) had a final score ≤ 5. Of the 23 papers, four (17.39%) received a maximum score of 9, whereas six studies (26.09%) received a score of 8, obtaining high quality scores in the three parameters. Among the 23 studies, the outcome parameter was the one with the lowest overall score, with 11 papers (47.82%) receiving only 1 point out of a total possible three points. Table 2 presents this information in detail.

**Table 2:** Outline of quality assessment: appraisal of items of the Newcastle-Ottawa Scale by researcher 1 (R1) and researcher 2 (R2).

	**Selection**		**Comparability**		**Outcome**		**Total score**	**Quality**
**Study**	**R1**	**R2**	**R1**	**R2**	**R1**	**R2**		
Banducci et al. (2017)	***	***	**	**	***	***	8*	High
Nousia et al. (2018)	****	****	**	**	***	*	9*	High
Chambon et al. (2014)	***	***	**	**	***	***	8*	High
Kurz et al. (2008)	****	****	**	*	*	*	7*	High
Aramaki and Yassuda (2011)	**	*	*	**	***	***	6*	High
Giovagnoli et al. (2017)	****	***	**	**	***	***	9*	High
Savulich et al. (2017)	****	****	**	**	*	**	7*	High
Neely et al. (2014)	****	****	**	**	*	*	7*	High
Legault et al. (2011)	***	***	**	**	*	*	6*	High
Lima-Silva et al. (2010)	***	***	*	*	*	*	5*	Low
Ball et al. (2002)	***	***	**	**	***	***	8*	High
Jean et al. (2014)	****	****	*	*	**	**	7*	High
Carvalho et al. (2009)	***	**	**	**	*	*	6*	High
Fandakova et al. (2012)	***	**		**	*	*	4*	Low
Belleville et al. (2006)	***	****	**	**	*	*	6*	High
Willis et al. (2006)	***	****	**	**	***	***	8*	High
Gross and Rebok (2011)	***	**	**	**	***	***	8*	High
McDougall et al. (2018)	****	****	**	**	***	***	9*	High
da Silva and Yassuda (2009)	***	***	**	**	*	*	6*	High
Langbaum et al. (2009)	***	***	**	**	*	***	6*	High
Kinsella et al. (2016)	****	****	**	**	***	***	9*	High
González-Palau et al. (2014)	***	***	*	*	*	*	5*	Low
Zimmermann et al. (2016)	***	***	**	*	***	***	8*	High

*A study can be awarded with stars for each numbered item within the categories according by Newcastle-Ottawa Scale (NOS)*.

**Figure 2:**
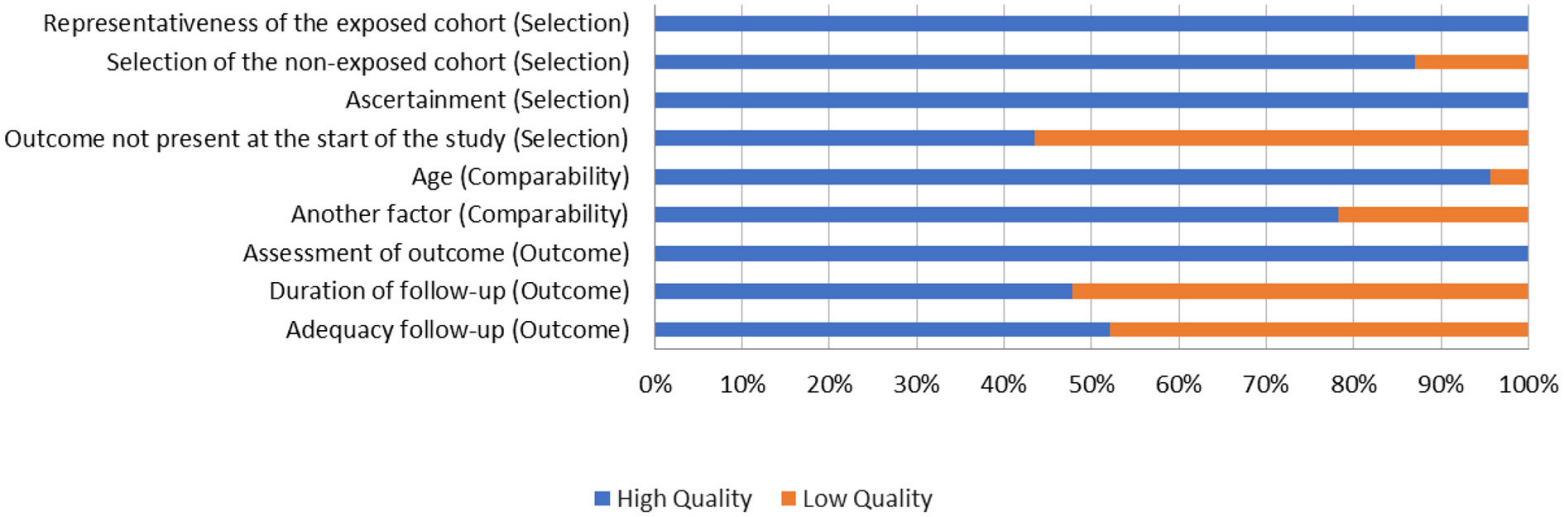
Methodological quality. Assessment of the included studies in each item of the Newcastle-Ottawa Scale, presented as a percentage.

Figure 2 shows the quality of the studies, organized by criteria and the percentage of studies that were scored in each of the criteria. For the item “Representativeness of the sample and assessment of the outcome,” all 23 studies gained points. Only 10 studies (43.48%) received points for the item “Outcome not present at the start of the study,” in which 13 studies (56.52%) had only healthy elderly individuals as the experimental group (i.e., they did not have initial cognitive difficulties, which could have influenced the beneficial effects of cognitive training). Only 11 studies (47.82%) scored on the items about cohort follow-up. The 12 studies (52.47%) that did not score in this regard either did not present a longitudinal study or they collected data within a period of <3 months after training, which was considered by the researchers as too short. For the item “Comparability,” the score was based on the age of the subjects in the experimental and control groups, in addition to another factor, such as education and the presence or absence of a clinical diagnosis and its type.

## Discussion

Despite being a widely studied topic, several empirical questions about interventions to improve memory during the aging process remain unanswered. The present systematic review sought to elucidate different designs of episodic memory training and their effectiveness in healthy elderly individuals with neurocognitive disorders. We identified 23 studies that met the inclusion criteria. The main variables that comprised the intervention protocols (i.e., characteristics of the sample, type of control group, mode of episodic memory training, and duration of training) were investigated, as well as the outcome variables, efficacy, and risk of bias.

The main results indicated effectiveness with regard to different forms of the cognitive training of episodic memory. These results are consistent with the literature on cognitive mechanisms that underlie this type of training. Episodic memory exercises can influence memory function at various levels, especially in the information coding and consolidation phases. Exercises that are performed before coding new information and during the period of memory consolidation (as opposed to exercises that are performed during memory coding) can improve the evocation of stored information (Loprinzi et al., 2017).

Another characteristic that was observed in the different studies is the effectiveness of training in healthy individuals and in subjects with dementia in the initial phase (Belleville et al., 2006; Chambon et al., 2014; Neely et al., 2014; Kinsella et al., 2016). According to Bahar-Fuchs et al. (2019), cognitive training can lead to overall improvements in cognitive functions in healthy individuals and, to a lesser extent, in subjects with some form of cognitive impairment. In the latter case, training specific functions, such as memory, more be more viable and beneficial.

The characteristics of the instruments that were used to evaluate memory in these studies are particularly important for detecting cognitive decline and assessing effectiveness of the intervention. Most of the studies used instruments based on word list tasks (e.g., RAVLT, HVLT-R, and CVLT), which comprise verbal exposure, retention, and the subsequent evocation of words by the examinee. Despite the fact that the use of this paradigm to assess episodic memory is canonical, notable is the lack of more ecological and contextual assessments that explore functionality of the patient in everyday situations.

The ecological assessment of episodic memory in elderly individuals may be useful for identifying subtle amnesic deficits that generally escape formal assessment, thus allowing investigations of the impact of the context where they occur. Studies suggest only low correlations between these tests and subjective complaints of memory function in daily life (Chaytor and Schmitter-Edgecombe, 2003; Reid and MacLullich, 2006). More ecological interventions and outcome variables may produce longer-lasting effects because of the influence on behaviors that can compromise functionality of the elderly and are frequent subjective complaints. da Silva and Yassuda (2009), Jean et al. (2014), and Neely et al. (2014) were among the few studies that employed ecological measures.

The ecological training of episodic memory favors the stimulation of information storage, the creation of an effect of spatial temporal contiguity, and the consolidation of lived experiences because this kind of training fosters mental storage and evokes a complex scene or event. According to Raffard et al. (2010), episodic and autobiographical memory deficiencies could be attributable to a specific deficit in construction of the scene, in which the impairment is not restricted only to the retrieval and integration of relevant spatial and temporal components but also to the manipulation of mental images that are evoked from personal life situations.

Notable differences were observed between the various forms of cognitive training and the experimental methods that were used. Consistent with the observations of Bahar-Fuchs et al. (2019), although the inclusion and exclusion criteria were useful, the remaining studies varied with regard to the form of stimulus between the experimental and control groups, the total investigated population, and clinical condition of the subjects, which likely influenced the findings.

### Methodological quality

Although we classified most of the studies as having a high risk of bias in at least two domains, our approach to classifying studies as high and low quality with regard to the risk of bias for the purpose of analyzing subgroups and classifying evidence has been relatively with low scores.

The low methodological quality of some of the studies for some of the criteria limits our ability to assess the base of evidence in the literature in this field. The quality of most of the studies of cognitive training interventions that were included in this review may have several risks of bias, particularly because of insufficient details with regard to heterogeneity of the instruments that were used to measure effectiveness, the variability of the interventions, and the lack of follow-up.

Although we classified all of the experimental interventions that were employed in the studies, they were clinically heterogeneous. Some of the interventions targeted only one cognitive domain, but some evaluated other domains simultaneously. We observed the diverse use of paper and pencil forms, training using computerized platforms, and ecological activities. Some interventions focused mainly on simple exercises, whereas others used a series of learning and performance strategies. The configurations (e.g., frequency and duration of the sessions) of the interventions were also diverse, and some were delivered at home, whereas others were delivered in the clinical settings.

In the reviewed studies, the results were evaluated according to different measures. In many cases, however, insufficient details were provided to determine which exact measure was used. When the studies reported individual subtest scores from test batteries or global indices, we considered each subtest as a measure of episodic memory. In some cases, the studies used unpublished tests that were developed specifically for the purposes of the particular study. This heterogeneity in the field reveals the plurality of approaches and measures, but it compromises the synthesis and integration of information.

### Research limitations

According to the analysis of the studies (Table 1), the heterogeneity of the characteristics of the studies hampers generalization of the results, such as (1) gender differences between studies (although all of the studies included both sexes, the female gender prevailed), (2) studies without a control group in their design (Aramaki and Yassuda, 2011), (3) variability of the training duration, especially the number of sessions and stimulation time in each session, varying in 10 different categories based on duration, and the time varying in seven different categories, (4) modality of the forms of intervention, and (5) variety of the instruments used, which varied among four different categories according to the number of times the instrument was used among the different studies. The analysis of the effectiveness of cognitive training can be influenced by any of these factors.

Although the studies met the inclusion and exclusion criteria, two of these criteria limited the analysis of some of the research variables. Although cohort studies offer a broader picture of comparisons between groups, the inclusion of clinical cases would provide an in-depth view of a specific pathology and indicate how cognitive training can help patient in these cases. Additionally, although the focus of the analysis was on episodic memory, other cognitive processes, such as attention and executive function, are involved in the consolidation and evocation of information. Therefore, one suggestion would be to observe how episodic memory training can favor this type of memory.

### Practical and theoretical implications

The present results rise the importance of cognitive training and socialization among the elderly, which can mitigate the manifestation of dementia and consequently the negative impact on daily life at the social, psychological, and economic levels (Kurz et al., 2008; Aramaki and Yassuda, 2011; Neely et al., 2014).

From a theoretical perspective, the present results are consistent with the literature. Cognitive training is based on the process of brain plasticity, stimulating changes in neural networks and favoring the cognitive functions that are stimulated. When considering the different intervention methods, one must consider the specific type of training with current cognitive status of the subject. For example, new learning skills and delayed recall should be trained in elderly individuals with early-stage AD or vascular dementia (Bahar-Fuchs et al., 2019).

## Conclusion

Several systematic reviews with and without meta-analyses have evaluated the effectiveness of cognitive training for the elderly. However, few studies have mapped the literature with regard to the cognitive training of episodic memory. The present findings indicate that our general and specific objectives were achieved. The results demonstrate the benefits of cognitive training and importance of performing further studies of new forms of care for the elderly.

Future studies should investigate the impact of clinical aspects at different levels: such as psychological, functional and physiological on episodic memory training. It is important to emphasize that memory is not an isolated process, as it is mutually influenced by other cognitive processes such as attention and executive functions, which also need to be investigated together in a global analysis. In addition, new review studies may expand the findings of the present study comparing the kind of syndromes and populations of different ages, cause deficits in episodic memory are not restricted to the elderly.

## Data availability statement

The raw data supporting the conclusions of this article will be made available by the authors, without undue reservation.

## Author contributions

CN and JL-F: conceived, supervised the review, and wrote the manuscript. AM, LL, and CN: carried out the review and wrote the manuscript. All authors contributed to the article and approved the submitted version.

## Funding

This study was financed in part by the Coordenação de Aperfeiçoamento de Pessoal de Nível Superior (CAPES), Brazil (Finance Code 001), Fundação de Amparo à Pesquisa do Estado do Rio de Janeiro (FAPERJ), and a scholarship from Concelho Nacional de Desenvolvimento Científico e Tecnológico (CNPq).

## Conflict of interest

The authors declare that the research was conducted in the absence of any commercial or financial relationships that could be construed as a potential conflict of interest.

## Publisher's note

All claims expressed in this article are solely those of the authors and do not necessarily represent those of their affiliated organizations, or those of the publisher, the editors and the reviewers. Any product that may be evaluated in this article, or claim that may be made by its manufacturer, is not guaranteed or endorsed by the publisher.
